# Intestinal Microbiota Succession and Immunomodulatory Consequences after Introduction of *Lactobacillus reuteri* I5007 in Neonatal Piglets

**DOI:** 10.1371/journal.pone.0119505

**Published:** 2015-03-16

**Authors:** Chengli Hou, Hong Liu, Jiang Zhang, Shihai Zhang, Fengjuan Yang, Xiangfang Zeng, Philip A Thacker, Guolong Zhang, Shiyan Qiao

**Affiliations:** 1 State Key Laboratory of Animal Nutrition, China Agricultural University, No. 2, Yuanmingyuan West Road, Beijing 100193, China; 2 Department of Animal and Poultry Science, University of Saskatchewan, Saskatoon, Canada; 3 Department of Animal Science, Oklahoma State University, Stillwater, Oklahoma, United States of America; German Institute of Human Nutrition Potsdam-Rehbrücke, GERMANY

## Abstract

Seventy-two, suckling piglets, obtained from 9 litters standardized to 8 piglets, were assigned to 1 of 3 treatments (n = 24) to compare short-term, early administration with intermittent, longer-term administration of *Lactobacillus reuteri* I5007. The treatments were a control (given a placebo of 0.1% peptone water from day 1 to 5) or treatments in which 1.7 × 10^10^ CFU *L*. *reuteri* was administrated either daily for 4 days starting on day 1 or every 4th day from day 1 to 17. Five piglets per treatment were killed at 3 time points (day 7, 14 and 21). Denaturing Gradient Electrophoresis of ileal digesta revealed an increase in the presence of *L*. *reuteri* I5007 and *Clostridium lentocellum* (on day 14 and 21) in the every 4th-day treatment and *Actinobacillus porcinus* (on day 7 and 14) in both *L*. *reuteri* treatments, while reducing the abundance of *E*. *coli* on day 21 in the every 4th-day treatment. Real-time qPCR of ileal digesta showed an increase in *Bifidobacterium* spp. on day 14 for both *L*. *reuteri* I5007 treatments. An increase in the concentration of lactic acid and a lower pH was observed in the first 4-day treatment on day 7 and the every 4^th^ day treatment on day 14. The relative abundance of mRNA for TGF-β was increased while that for IFN-γ was decreased in the mesenteric lymph nodes of piglets treated with *L*. *reuteri* every 4^th^ day. In conclusion, early intervention with *L*. *reuteri* increases the presence of beneficial bacteria and decreases the presence of undesirable microbes in the lower gastrointestinal tract. The changes appear to be mediated by altering the intestinal pH through lactic acid production resulting in favorable bacterial species colonization. A prolonged duration of treatment (i.e. every 4^th^ day) would appear to be superior to treatment only during the first 4 days.

## Introduction

The presence and composition of the microbiota in the intestine in early life makes a key contribution to the establishment of gut homoeostasis and maturation of the immune system [1–3]. Microbial colonization is a complex process made more challenging by the low diversity of intestinal microbes and the under developed immune system of neonates [4,5]. However, a large number of studies have demonstrated that early intervention with desirable microbes may help to establish a stable bacterial ecology and enhanced immunological development [5–8]. Thus, strategies resulting in the establishment of an effective gut ecosystem should be implemented in early life.

Lactobacilli are normal inhabitants of the gastrointestinal tract in both humans and animals and have been intensively researched during the past several decades for their specific probiotic properties and effects on the intestinal microbiota [9,10]. *Lactobacillus reuteri* (*L*. *reuteri*) I5007, initially known as *L*. *fermentum* I5007, was isolated in our laboratory from the colonic mucosa of healthy, weaned piglets. It has been shown to possess many excellent probiotic characteristics, including digestive enzyme tolerance, high adhesion to Caco-2 cells, competitive exclusion against pathogen invasions and alleviation of weaning stress in piglets [11–13].

Research has shown that oral administration of *L*. *reuter*i I5007 can improve performance, reduce the incidence of diarrhea and improve the intestinal health of suckling piglets [14]. However, detailed knowledge about the mechanism through which *L*. *reuteri* mediates its effects is not yet available. In addition, there is limited information available about the most appropriate dosing regimen. The only published research in which *L*. *reuteri* has been administered to suckling piglets involved daily administration for the first 14 days of life [14]. From a management perspective, this is very labor intensive. It is not known whether treatment for a shorter period of time or the use of an intermittent treatment regimen can also be effective in establishing an effective gut ecosystem.

The aim of the current study was to investigate the effects of *L*. *reuteri* I5007 administration on the microbial populations in the lower gastrointestinal tract, short chain fatty acid (SCFA) and pH levels in the colon, and cytokine and transcription factor expression in the mesenteric lymph nodes of suckling pigs. In addition, a comparison was made of short-term administration (i.e. daily for 4 days starting on day 1) with longer term intermittent administration (i.e. every 4th day from day 1 to 17). Our hypothesis was that intermittent treatment for a longer period of time would be superior to short-term daily administration.

## Materials and Methods

### Ethics statement

The procedures used in this experiment were approved by the China Agricultural University Institutional Animal Care and Use Committee (Beijing, China).

### Growth and storage conditions for *L*. *reuteri* I5007

*L*. *reuteri* I5007 was cultivated in sterile De Man Rogosa Sharpe media for 24 h at 37°C in an anaerobic environment, followed by centrifugation at 5000 x g for 10 min at 4°C. The cells were re-suspended in reconstituted skim milk (20% wt/vol) and immediately freeze-dried. The freeze-dried powder containing 1.7 x 10^10^ colony forming units (CFU)/g was stored in sealed packets at a temperature of 4°C until needed.

### Animals and treatments

Nine, Large White x Landrace, crossbred sows were artificially inseminated by one Duroc boar to minimize the genetic variation among their off-spring. The sows were allowed to farrow in concrete-floored farrowing pens at the China Agricultural University Swine Research Facility located in Beijing, China (non-SPF facility) and litter size was standardized to 8 piglets. The piglets which remained in the litter were selected so that each litter was balanced for average weight and sex. All surplus piglets were cross fostered to sows not on the experiment.

One day 1, the litters were allocated to 1 of 3 treatments with 3 litters of 8 piglets assigned to each treatment (n = 24). The treatments comprised a control treatment (piglets given a placebo of 4 mL of 0.1% peptone water on days 1 to 5) or two experimental treatments in which 1.7 × 10^10^ CFU *L*. *reuteri* was administrated either daily from day 1 to 5 or every 4th day from day 1 to 17 (i.e. days 1, 5, 9, 13 and 17). One gram of *L*. *reuteri* was dissolved in 4 mL of 0.1% peptone water for oral administration to each pig using a 5 mL syringe.

Piglets were given iron supplementation and had their teeth and tails cut within the first three days life, but had no access to creep feed during the experiment and therefore relied on sow’s milk as their sole source of nutrients. On days 7, 14 and 21, five piglets closest to the average weight for each treatment (regardless of litter) were euthanized with electricity, and the luminal contents from the ileum and proximal distal colon were collected and then stored at -80°C. On day 21, mesenteric lymph node samples, were excised from the ileum of each piglet, and stored at -80°C until total RNA was extracted.

### Determination of colonic short chain fatty acid (SCFA) concentrations and pH

The SCFA in the colonic digesta were measured using a gas chromatographic method following the preparation procedures of Franklin *et al*. [15] using a HP 6890 Series Gas Chromatograph (Hewlett Packard, Palo Alto, CA) equipped with a HP 19091N-213 column with 30.0 m × 0.32 mm i.d. (Agilent, Palo Alto, CA). The pH of the digesta samples was measured using a digital IQ150 pH meter (Spectrum, Chicago, IL).

### DNA isolation

To improve the amount and quality of DNA, the luminal samples (approximately 0.2 g) were beaten with glass beads, using a Mini Bead Beater (BioSpec Products, Bartlesville, OK) according to Zoetendal *et al*. [16]. The DNA was then extracted using a QIAamp Stool Mini-Kit (Qiagen, Hilden, Germany) following the manufacturer’s protocol. The concentration of extracted DNA was measured using a NanoDrop Spectrophotometer (P330, Implen, Germany).

### Denaturing gradient gel electrophoresis (DGGE)

The luminal contents (ileal and proximal colon) from individual piglets (n = 4 per treatment) were used to analyze the profile of the gut microbiota using DGGE methodology. Due to lack of space on the gels, only 4 piglets per treatment were analyzed. The 4 piglets chosen for DGGE analysis were randomly selected from the 5 pigs slaughtered at each time period.

Two sets of primers (F-968-GC: 5′-CGC CCG GGG CGC GCC CCG GGC GGG GCG GGG GCA CGG GGG GAA CGC GAA GAA CCT TAC-3′ and R-1401: 5′-CGG TGT GTA CAA GAC CC-3′) were used for PCR amplification targeting the V6–V8 regions of the 16S rRNA gene according to the procedures of Nübel *et al*. [17]. PCR was performed with a GoTaq Hot Start Polymerase Kit (Promega, Madison, WI). The PCR mixture (50 μL) contained 2 U GoTaq Hot Start Polymerase, 10 μL of 5 x Colorless GoTaq^®^Flexi Buffer, 4 μL of deoxynucleoside triphosphate, 2 μL of 25 mmol/L MgCl_2_, 0.5 pmol/L dNTPs, and 1 μL of DNA template. The final volume was adjusted to 50 μL with sterile deionized water.

Amplification programs were set using a Bio-Rad Thermocycler (Bio-Rad, Hercules, CA) with an initial denaturation at 94°C for 3 min followed by 35 cycles consisting of denaturation at 94°C for 30 s, annealing at 56°C for 20 s, and extension at 68°C for 40 s. The final extension was set at 68°C for 7 min. The amplicons were checked by electrophoresis on 1% (wt:vol) agarose gel in 1% SYBR Green I (Sigma, St, Louis, MO) to verify that a single product of the expected size was obtained.

The target amplicons were separated by DGGE. Electrophoresis was performed on 8% (wt:vol) polyacrylamide gels using a vertical gradient of denaturants between 45 and 58% (a 100% denaturing solution was defined as 40% (vol:vol) formamide and 7 M urea). For each amplicon, the same concentration of DNA was loaded in one lane of the gel.

Electrophoresis was initiated by pre-running for 10 min at 200 V and then for 16 h at 85 V in 0.5 x Tris-acetate-EDTA buffer at a constant temperature of 60°C in a DCode System Apparatus (Bio-Rad, Hercules, CA). The DGGE gels were stained by SYBR Green I (Sigma, St, Louis, MO) while being agitated for 15 min in an orbital shaker (Qilinbeier, Haimen, China) at room temperature, and then photographed with a Gel Doc XR^+^ System (Bio-Rad, Hercules, CA), and analyzed with Quantity One Basic 4.6.6 (Bio-Rad, Hercules, CA).

The total number of bands and the Shannon-Weaver index were used to quantify microbial diversity as described by Gafan *et al*. [18]. Predominant bands (five bands) in pre-run DGGE gels were excised, purified and amplified. Amplicons of the V6–V8 regions of 16S rRNA from the pure strain of *L*. *reuteri* I5007 were prepared by mixing equal amounts and used as the marker for gel to gel comparison.

Bands of interest were excised from the original gels and their DNA fragments were re-amplified under the same conditions using corresponding primers without the GC Clamp. The PCR products were purified and concentrated using a Wizard SV Gel and PCR Clean-Up System (Promega, Madison, WI) and then transformed into *E*. *coli* JM109 using the pGEM-T vector system (Promega, Madison, WI) according to the manufacturer’s instructions. The transformants were grown on Luria-Bertani agar in the presence of 100 μg/mL ampicillin and 20 μg/mL X-Gal. The positive recombinants (3 clones on each band) were screened and identified by PCR and sent for sequencing (Shanboyuanzhi Service, Beijing, China). Homology searches were performed using the BLAST program in the NCBI database (http://blast.ncbi.nlm.nih.gov/).

### Nucleotide sequence accession numbers

The sequences presented in Table 1 were deposited in the EMBL databases under the accession numbers HF952840-HF952849, HF952850-HF952858, and HF952859-HF952864 for the ileal profile, colonic profile and the marker respectively.

**Table 1:** Closest relatives to sequences excised from the DGGE profile obtained from ileal and colonic digesta (band numbers refer to Figs. 1 and 2).

Ileum			Colon		
Band Number	Closest cultured relative	% Similarity	Band Number	Closest cultured relative	% Similarity
1	*Clostridium perfringens*	100	1	*Clostridium glycolicum*	95
2, 3	*Clostridium lentocellum*	93	2	Uncultured bacterium	99
4	*Clostridium* spp.	99	3, 4	*Clostridium clostridioforme*	99
5	*Streptococcus henryi*	92	5	Uncultured bacterium	95
6, 7	*Actinobacillus porcinus*	99	6	*Oscillibacter valericigenes*	90
8	*Escherichia coli*	99	7	*Bacillus weihenstephanensis*	92
9	*Lactobacillus vaginalis*	99	8	*Clostridium aldenense*	94
10	*Lactobacillus reuteri* I5007	99	9	*Lactobacillus* spp.	99
11	Uncultured bacterium	99	10	*Lactobacillus reuteri* I5007	99

### Quantification of targeted genus groups in the luminal contents by real-time qPCR

The abundance of targeted bacteria in the intestinal luminal contents were analyzed by real-time qPCR. Concentrations of *Bifidobacterium* spp., *Lactobacillus* spp. and total bacteria were determined in both ileal and colonic digesta on days 7, 14 and 21. The primers used are listed in Table 2.

**Table 2:** Primer sequences used in the study.

Primer	Orientation	Sequence 5′-3′	*R*	Reference
*Bifidobacterium* spp.	Bif164F	GGGTGGTAATGCCGGATG	0.99	[19]
	Bif662R	CCACCGTTACACCGGGAA		
*Lactabacillus* spp.	LacF	AGCAGTAGGGAATCTTCCA	0.99	[20]
	LacR	CACCGCTAC ACATGGAG		
Total bacteria	Uni331F	TCCTACGGG AGGCAGCAGT	0.99	[21]
	Uni797R	GGACTACCAGGGTATCTATCCTGTT		
IL-17	Sense	CAGGTCATCACCATCGGCAACG	0.99	[22]
	Anti-sense	GACAGCACCGTGTTGGCGTAGAGGT		
IL-4	Sense	TTGCTGCCCCAGAGAAC	0.99	[22]
	Anti-sense	TGTCAAGTCCGCTCAGG		
IL-10	Sense	ATGGGCGACTTGTTGCTGAC	0.99	[23]
	Anti-sense	CACAGGGCAGAAATTGATGACA		
IL-1β	Sense	CCTCCTCCCAGGCCTTCTGT	0.98	[24]
	Anti-sense	GGGCCAGCCAGCACTAGAGA		
IL-6	Sense	TGGATAAGCTGCAGTCACAG	0.99	[25]
	Anti-sense	ATTATCCGAATGGCCCTCAG		
Foxp3	Sense	GGTGCAGTCTCTGGAACAAC	0.99	[26]
	Anti-sense	GGTGCCAGTGGCTACAATAC		
TGF-β	Sense	CAGAGAGGCTATAGAGGGTT	0.98	[27]
	Anti-sense	TGTCTAGGCTCCAGATGTAG		
TNF-α	Sense	CCCAAGGACTCAGATCATCG	0.99	[24]
	Anti-sense	ATACCCACTCTGCCATTGGA		
IFN-γ	Sense	GCAAGTACCTCAGATGTACC	0.99	[27]
	Anti-sense	TGGCCTTGGAACATAGTCTG		
IL-12p40	Sense	GGAGTATAAGAAGTACAGAGTGG	0.99	[22]
	Anti-sense	GATGTCCCTGATGAAGAAGC		

Standard curves were constructed using the PCR products of *Lactobacillus acidophilus* ATCC 4356, *Bifidobacterium longum* ATCC 15707 and *Escherichia coli* ATCC K88. *Bifidobacterium longum* ATCC 15707 was grown in trypticase-peptone-yeast extract-glucose media and *L*. *acidophilus* ATCC 4356 was grown in De Man Rogosa Sharpe broth at 37°C under aerobic conditions. *Escherichia coli* K88 (ATCC K88) was grown in Luria-Bertani media at 37°C under aeration.

Three serial 10-fold dilutions of extracted DNA samples were applied to qPCR in an Applied Biosystems 7500 Real-Time PCR System (Applied Biosystems, Singapore) using the SYBR Green PCR Master Mix (Promega, Madison, WI). Amplification was performed in triplicate reactions for each bacterial genus within each sample. For amplification, 20 μL of a final volume containing 2 x SYBR Green PCR Master Mix (Promega, Madison, WI), 15 pmol of each primer, and 2 μL of DNA template was used. PCR protocols for the above bacterial genus were obtained by a linear regression curve and then log copy numbers were plotted against *C*_T_ values. The standard curves had correlation coefficients of 0.994 for *Bifidobacterium*, 0.991 for *Lactobacillus*, and 0.993 for total bacteria. The linear range of the standard curves for *Bifidobacterium* spp., *Lactobacillus* spp., and total bacteria was the same and ranged from 2 to 9 log (copy/g).

### Real-time PCR analysis of the targeted genes in the mesenteric lymph nodes

Total RNA was isolated from the mesenteric lymph nodes using a RNeasy Mini Kit (Qiagen, Hilden, Germany). The purity was determined by the ratio of A_260_:A_280_ using a NanoDrop Spectrophotometer (P330, Implen, Germany) and then the quality was checked with 1% agarose gel electophoresis following the procedures outlined by Aranda *et al*. [28]. The extracted RNA was converted into first-strand cDNA by reverse transcription of 1 μg of total RNA using a PrimeScript 1st Strand cDNA Synthesis Kit (Takara, Dalian, China) according to the manufacturer’s protocol and stored at -80°C. Real-time PCR was performed on an Applied Biosystems 7500 Real-Time PCR System (Applied Biosystems, Singapore) using SYBR Green PCR Master Mix (Takara, Dalian, China). All reactions were run in triplicate. The mRNA expression is expressed as the ratio between the targeted gene and β-actin gene expression.

### Data analysis

Statistical comparisons of measured indices among the three treatments were performed by Analysis of Variance followed by Student Neuman Keuls’s multiple-comparison test using the Statistical Analysis Systems Statistical Software Package Version 8.02 (SAS Institute, Cary, NC). Individual pig served as the experimental unit. Differences at *P* ≤ 0.05 were considered significant.

## Results

### Analysis of the microbiota populations using PCR-DGGE analysis

**Fig 1:**
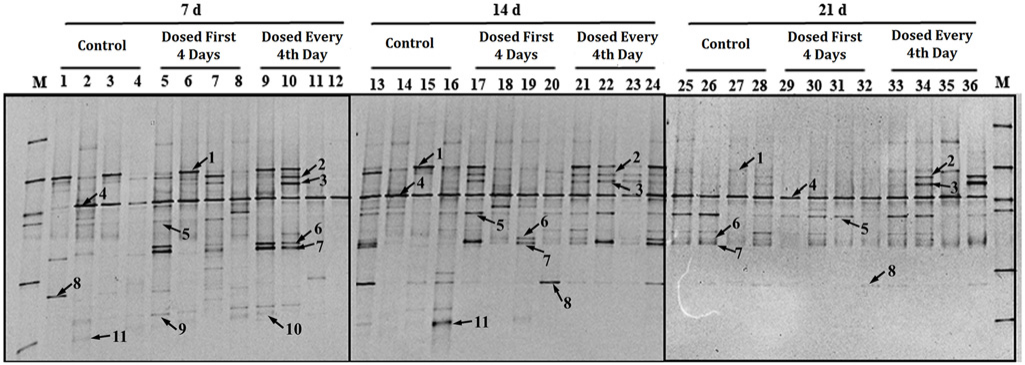
DGGE profile of the PCR products of the V6 to V8 regions of 16S rRNA obtained from ileal digesta of piglets on day 7, 14 and 21. n = 4 for each treatment. Lane M, marker. Arrows (1 to 11) indicate excised bands that were re-amplified and sequenced. Lane 1, *Clostridium perfringens*; lanes 2, 3, *Clostridium lentocellum*; lane 4, *Clostridium* spp.; lane 5, *Streptococcus henryi*; lanes 6, 7 *Actinobacillus porcinus*; lane 8, *Escherichia coli*; lane 9, *Lactobacillus vaginalis*; lanes 10, *Lactobacillus reuteri* I5007; lane 11, Uncultured bacterium. Bacteria 9, 10 in the 14 day samples and 9, 10, and 11 in the 21 day samples were unable to be detected in the DGGE profile.

**Fig 2:**
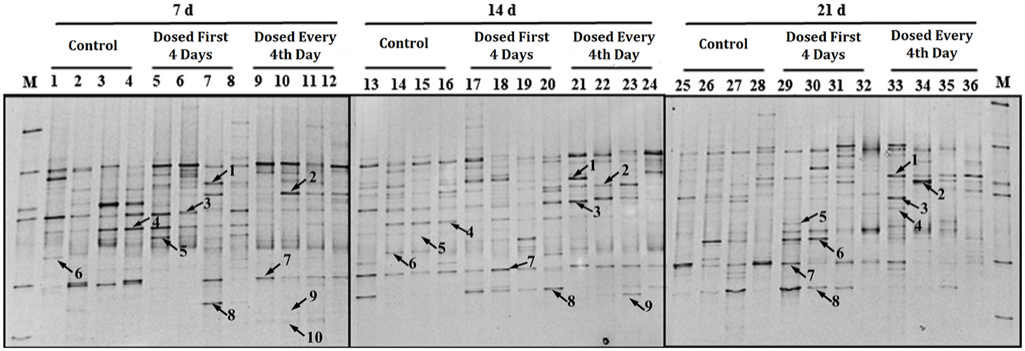
DGGE profile of PCR products from the V6 to V8 regions of 16S rRNA obtained from colonic digesta of piglets on day 7, 14 and 21. n = 4 for each treatment. Arrows (1 to 10) indicate excised bands that were re-amplified and sequenced. Lane M, marker. Lane 1, *Clostridium glycolicum*; lane 2, Uncultured bacterium; lanes 3, 4. *Clostridium clostridioforme*; lane 5, Uncultured bacterium; lane 6, *Oscillibacter valericigenes*; lane 7, *Bacillus weihenstephanensis*; lane 8, *Clostridium aldenense*; lane 9, *Lactobacillus* spp.; lane 10, *Lactobacillus reuteri* I5007. Bacteria 10 in the 14 day and bacteria 9, 10 in the 21 day sample were not unable to be detected in the DGGE profile.

To compare the different species of bacteria present in the gastrointestinal tract among the treatments, the V6–V8 regions of 16S rRNA PCR amplicons were analyzed and are presented in Figs. 1 and 2 respectively. Each profile had thirty-six sample lanes. The bands marked in the profiles represent microbial populations of interest in the luminal contents, and their presence or absence, reflect changes in microbial community succession.

From the profiles (Fig. 1), we observed that *L*. *reuteri* I5007 administration successfully altered the microbiota composition of the ileal digesta. The main effects of *L*. *reuteri* I5007 on the bacterial communities were in increasing the abundance of *Clostridium lentocellum* (on day 14 and 21) in the every 4th-day treatment and *Actinobacillus porcinus* (on day 7 and 14) in both *L*. *reuteri* treatments, while reducing the relative abundance of *E*. *coli* on day 21 in the every 4th-day treatment. *Lactobacillus vaginalis* was observed to increase in abundance with both *L*. *reuteri* I5007 treatments on day 7 while the *L*. *reuteri* I5007 strain was also increased at this time point compared with the control treatment. We also observed that *Clostridium perfringens*, *Clostridium* spp., *Streptococcus henryi* and *Escherichia coli* were the predominant inhabitants in the ileal contents during the experiment, with an increase in the proportions of *Streptococcus henryi* on day 14 and 21 while there was a reduction in *Clostridium perfringens* on day 21.

Compared with the bacterial composition in the ileal contents, the bacteria in the colonic samples were more diversified (Fig. 2, Tables 3 and 4). By day 21, there was approximately a 2-fold difference in the number of bands found in the colonic digesta compared with the ileal digesta. In addition, the Shannon diversity index was higher in the colonic digesta than in the ileal digesta. At day 21, the number of bands in the ileal digesta in the first 4-day treatment was significantly lower than for the other two treatments. For colonic digesta, there was no difference in the number of bands or in the Shannon diversity index as a result of treatment.

**Table 3:** Effect of *L*. *reuteri* I5007 on the number of bands and microbial diversity in ileal digesta.

	Control	Dosed first 4 days	Dosed every 4th day	SEM*	*P* value
Day 7					
Number of bands	11.25	13.75	10.50	1.34	0.25
Shannon diversity	2.35	2.54	2.23	0.13	0.28
Band similarity index	49.08	54.25	50.62	4.50	0.71
Day 14					
Number of bands	13	12.75	11.75	0.94	0.62
Shannon diversity	2.47	2.50	2.39	0.08	0.61
Band similarity index	56.57	56.55	60.62	3.69	0.68
Day 21					
Number of bands	9.75 ^a^	6.25 ^b^	9.75 ^a^	0.92	0.04
Shannon diversity	2.15^a^	1.72^b^	2.18^a^	0.12	0.05
Band similarity index	47.45	58.10	63.03	4.36	0.06

*SEM, Standard Error of the Mean, n = 4 for each treatment.

^a, b^Means within a row with same or no superscript do not differ (*P>* 0.05).

**Table 4:** Effect of *L*. *reuteri* I5007 on the numbers of bands and microbial diversity in the colonic digesta.

	Control	Dosed first 4 days	Dosed every 4th day	SEM*	*P* value
Day 7					
Number of bands	11.23	13	13	0.92	0.35
Shannon diversity	1.69	2.73	2.65	0.22	0.31
Band similarity index	57.55	63.85	60.78	3.82	0.52
Day 14					
Number of bands	14.75	17	13.5	1.39	0.25
Shannon diversity	2.60	2.75	2.40	0.09	0.06
Band similarity index	63.55	64.73	57.88	3.41	0.34
Day 21					
Number of bands	15.5	14.5	17.5	1.17	0.23
Shannon diversity	2.59	2.49	2.90	0.17	0.25
Band similarity index	49.83	39.67	45.60	4.25	0.26

*SEM, Standard Error of the Mean, n = 4 for each treatment.

*Clostridium clostridioforme*, *Bacillus weihenstephanensis* and *Clostridium glycolicum* were the predominant species observed in the colon throughout the experiment, which was different from the profile in the ileum. Numbers of *Clostridium glycolicum* were increased with both *L*. *reuteri* I5007 treatments, while the *Clostridium clostridioforme* like phenotype was decreased in proportion in the every 4th-day treatment on day 7 and 21. Numbers of *Clostridium aldenense* were decreased on day 21 in the every 4th-day treatment. An increase in the numbers of *Clostridium aldenense* in the first 4 day treatment was observed compared with the other two treatments on day 14. The *L*. *reuteri* I5007 strain (99% identity) was positively detected on day 7 in both ileal and colonic profiles for both *L*. *reuteri* I5007 treatments.

### Quantification of targeted bacteria in ileal and colonic digesta by qPCR

**Fig 3:**
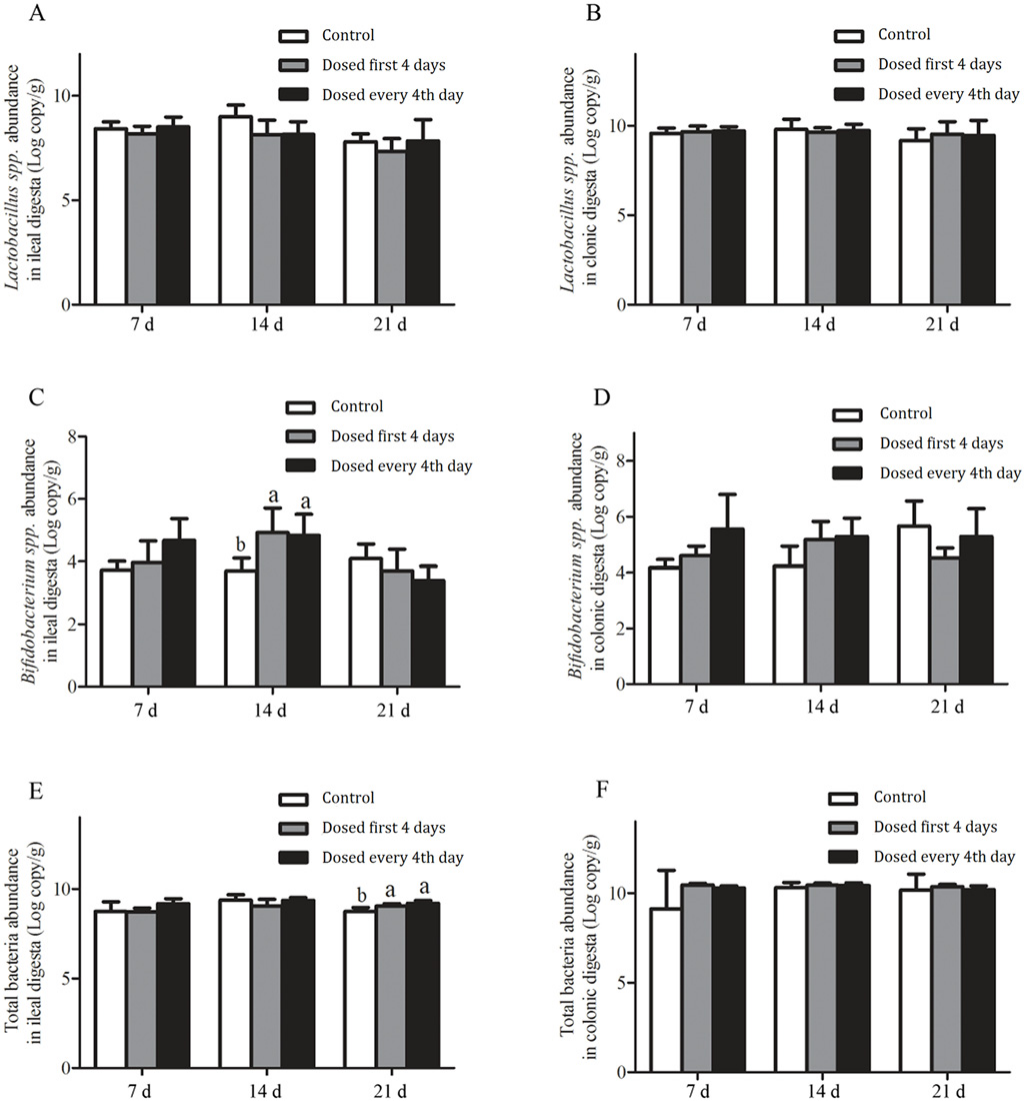
Abundance of *Lactobacillus* spp.(A, B), *Bifidobacterium* spp. (C, D) and total bacteria (E, F) analyzed by RT-qPCR in ileal and colonic digesta obtained from piglets on d 7, 14 and 21. Values are means ± SD (n = 5). Bars with different letters differ, *P* < 0. 05.

Analysis of targeted bacteria by qPCR revealed that the abundance of *Lactobacillus* spp. did not change with either *L*. *reuteri* I5007 treatment in either ileal or colonic digesta (*P* > 0.05. Fig. 3). Numbers of *Bifidobacterium* spp. in the ileal digesta were significantly increased with both *L*. *reuteri* I5007 treatments compared with the control treatment on day 14 (*P* < 0.05), while the numbers of total bacteria also showed a similar trend (*P* < 0.05) in the ileal digesta on day 21.

### pH and short chain fatty acid concentrations in colonic digesta

On day 7, a lower pH in the colonic digesta, accompanied by an increased concentration of lactic acid was observed in the first 4 days treatment compared with the control treatment (*P* < 0.05. Table 5) while no significant differences were observed between the control and the every 4th-day treatment (*P* > 0.05). On day 14, the pH in the colonic digesta of pigs in the every 4th-day treatment was significantly lower compared with the control treatment (*P* < 0.05), and lactic acid concentrations were observed to be significantly higher (*P* < 0.05). No significant differences were found between the control and the first 4 days treatment (*P* > 0.05). On day 21, no differences were observed among the three treatments in either pH or lactic acid concentrations in the colonic digesta (*P* > 0.05). The concentrations of acetic acid, propionic acid and butyric acid were not altered by treatment on any day.

**Table 5:** Effects of *L*. *reuteri* I5007 on pH and short chain fatty acid concentrations (mmol/kg) in colonic digesta obtained from neonatal piglets on days 7, 14 and 21.

	Control	Dosed first 4 days	Dosed every 4th day	SEM*	*P* value
Day 7					
pH	6.76^a^	6.15^b^	6.54^a^ ^b^	0.11	0.01
Lactic acid	2.01^b^	3.76^a^	2.83^a^ ^b^	0.45	0.05
Acetic acid	22.09	26.71	30.10	3.62	0.33
Propionic acid	0.75	0.67	0.65	0.16	0.89
Butyric acid	4.16	5.21	6.00	1.05	0.48
Day 14					
pH	6.69^a^	6.58^a^ ^b^	6.16^b^	0.13	0.03
Lactic acid	0.55^b^	0.79 ^a^ ^b^	1.03^a^	0.12	0.05
Acetic acid	19.45	28.72	19.31	3.30	0.11
Propionic acid	0.39	0.60	0.35	0.09	0.19
Butyric acid	2.69	2.54	2.24	0.48	0.79
Day 21					
pH	6.45	6.32	6.28	0.09	0.40
Lactic acid	1.59	1.92	2.36	0.57	0.65
Acetic acid	22.71	28.42	22.74	3.67	0.47
Propionic acid	0.74	0.72	0.65	0.16	0.92
Butyric acid	5.72	6.60	4.88	1.22	0.62

*SEM, Standard Error of the Mean, n = 5 for each treatment.

^a, b^Means within a row with same or no superscript do not differ (*P>* 0.05).

### Relative mRNA expression of cytokine and transcription factors in the mesenteric lymph node

**Fig 4:**
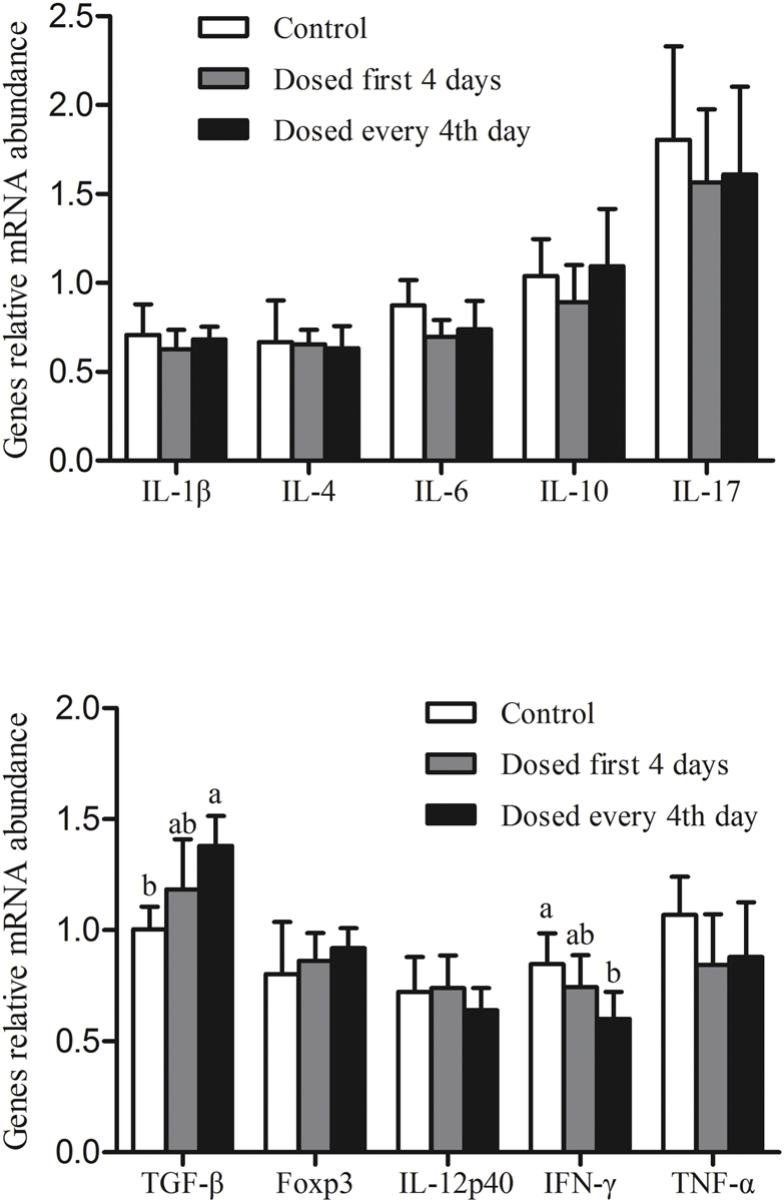
Effects of *L*. *reuteri* I5007 on relative cytokine and transcription factor expression in the mesenteric lymph node s (MLN) obtained from piglets and analyzed by RT-qPCR on d 21. Values are means ± SD (n = 5). Bars with different letters differ, *P* < 0. 05.

At the end of the experiment on day 21, the relative transcription levels of various cytokines and transcription factors in the mesenteric lymph node were analyzed using real-time qPCR (Fig. 4). The relative abundance of mRNA for TGF-β was increased while that for IFN-γ was decreased in the mesenteric lymph nodes of piglets treated with *L*. *reuteri* every 4^th^ day while the values for piglets treated with *L*. *reuteri* in the first 4-day treatment were intermediate to the other two treatments. mRNA levels for IL-1β, IL-4, IL-6 IL-10, IL-17, IL-12p40, TNF-α, and Foxp3 were unaffected by dietary treatment.

## Discussion

Immediately after birth, the gastrointestinal tract of neonates is involved in a process of colonization and succession by a complex and dynamic community of microbiota [29,30]. Accumulated evidence has demonstrated that early exposure with desirable microbiota followed by an appropriate colonization process in early life could alter the pattern of microbial succession as well as immunological maturation [31,32]. This experiment examined the impact of *L*. *reuteri* administration on the microbial populations in the gastrointestinal tract, VFA and pH levels in the colon, and cytokine and transcription factor expression in the mesenteric lymph nodes.

In order to function as a probiotic and have the ability to alter the microbial populations in the lower gastrointestinal tract, *L*. *reuteri* I5007 must be able to survive passage through the upper gastrointestinal tract and colonize in the lower intestinal tract. It did so successfully, as shown by it being positively identified in both the ileal and colonic digesta on day 7. However, we were unable to identify *L*. *reuteri* I5007 in either ileal or colonic digesta on day 14 and 21. This can be explained by the fact that bacterial populations can only be detected by DGGE when they comprise more than 1% of the total bacterial community [33]. The microbiota in the gut slowly build up after the birth and *L*. *reuteri* I5007 can temporarily dominate an unstable intestinal microbiota (on day 7). As the piglets age, the abundance of other gut bacteria increased. As a result, *L*. *reuteri* I5007 only comprised a small proportion of the gut bacteria community on days 14 and 21 and therefore could not be detected by DGGE.

Having proven that *L*. *reuteri* I5007 was able to survive passage through the upper gastrointestinal tract and colonize in the lower intestinal tract, the next step was to determine what effect it had on the numbers of beneficial bacteria in the lower intestinal tract. Accumulated studies have demonstrated that probiotic exposure could contribute to an increase in the numbers of Bifidobacteria and Lactobacilli in the luminal environment [34,35], and our experiment showed similar findings as evidenced by the increased abundance of *L*. *vaginalis* and *L*. *reuteri* I5007 shown by DGGE of ileal digesta while real-time qPCR of the ileal digesta showed an increase in the number of *Bifidobacterium* spp. on day 14 for both *L*. *reuteri* I5007 treatments.

The increased abundance of Lactobacilli and Bifidobacteria was accompanied by a decreased abundance of *E*. *coli* in the ileal digesta in the every 4th-day treatment throughout the experiment. Researchers have reported that *E*. *coli* are normal inhabitants in the intestine of neonates but can also act as opportunistic pathogens, and the reduction of these putrefactive bacteria may reduce the probability of pathophysiology disorders [36,37]. Based on above collective data we conclude that the exogenous strain of *L*. *reuteri* I5007 could temporarily dominate in the intestinal communities and manipulate the microbiota profile resulting in an increase in beneficial bacteria and a decrease in pathogenic bacteria.

It is reasonable to speculate that the alterations in the microbial community due to the exposure to *L*. *reuteri* I5007 are mediated by the reduced pH observed in the lower gastrointestinal tract as a result of *L*. *reuteri* I5007 administration. On day 7, a lower pH in the colonic digesta, accompanied by an increased concentration of lactic acid was observed in the first 4 days treatment compared with the control treatment. On day 14, the pH in the colonic digesta of pigs in the every 4th-day treatment was significantly lower compared with the control treatment and lactic acid concentrations were observed to be significantly higher. This theory is supported by a previous study which observed that high numbers of lactobacilli could shape the gastrointestinal niche by altering the environmental pH through lactic acid production and favorable bacterial species colonization [32].

Short chain fatty acids are the major products from hindgut fermentation processes and have been considered as contributing an important role in normal gut maintenance [3,38]. Our previous work [15] and that of others [39] indicated that propionic acid is usually the second most prominent VFA in colonic digesta. In the present experiment, the quantity of propionic acid in all samples was very low in comparison to acetic and butyric acid. We have no explanation for this apparent discrepancy.

An important finding of the present study was that the microbial communities found in the ileum were different from those seen in the colon. We observed that *Clostridium perfringens*, *Clostridium* spp., *Streptococcus henryi* and *Escherichia coli* were the predominant inhabitants in the ileal digesta while *Clostridium clostridioforme*, *Bacillus weihenstephanensis* and *Clostridium glycolicum* were the predominant species observed in the colon.

The major microorganisms detected in the present study are all the part of the normal microbiota of the digestive tract of piglets. *Clostridium* spp. is a broad genus, and are ubiquitous in the gastrointestinal tract [40]. In addition, previous studies have shown that the intestine of neonatal piglets is initially colonized by large numbers of *E*. *coli* and *Streptococcus* spp. [41], which become the dominant bacteria at the end of the first week of life and remain present for the entire suckling period [42]. We also found these strains in the ileum of neonatal piglets in the present study. *Bacteroides* and low densities of *Eubacterium*, *Fusobacterium*, and *Propionibacterium* are usually found in the suckling period [42,43], but they were not detected in the present study due to the limitations of the method used.

Not only did the most prevalent microbiota differ between the ileum and the colon but bacterial diversity varied as well. By day 21, there was approximately a 2-fold difference in the number of bands found in the colonic digesta compared with the ileal digesta. In addition, the Shannon diversity index was higher in the colonic digesta than in the ileal digesta. Konstantinov *et al*. [44] reported that a higher number of DGGE bands were observed in the colon (24.2 ± 5.5) than in the ileum (9.7 ± 4.2) of weaning piglets. The present study showed that the number of DGGE bands in the colon was lower than Konstantinov *et al*. [44] described. However, the number of bands was consistent with what Liu *et al*. [14] observed in neonatal piglets. Potential reasons for the discrepancy between Konstanintov *et al*. [44] and the results of the present study and that of Liu *et al*. [14] were that the samples were collected at different ages and the piglets were fed different diets.

Based on the findings reported above, it is evident that one mechanism through which *L*. *reuteri* I5007 functions is by increasing the presence of beneficial bacterial species in the lower gastrointestinal tract and decreasing the presence of undesirable microbes. We also studied the immunological consequences of early intervention with *L*. *reuteri* I5007.

A mature intestine harbors millions of commensal bacteria with thousands of species [45,46]. Thus, it is essential that the intestinal mucosa and epithelial cells develop an organized and elaborate mechanism for fighting pathogenic microbes. A beneficial microbial community could contribute to the development and maturation of the immune system [1]. Recently published data have proven that early exposure to beneficial microbial during the crucial stage of immune maturation is essential for the development of an effective immune system [2,7,47], and targeted microbes can be artificially manipulated to favor a beneficial gut colonization and also future immune system responses [6,48].

IFN-γ, which is commonly used as a marker for pro-inflammatory responses [49], was observed to be significantly reduced in the every 4th-day treatment compared with piglets in the control group. In one of our previous studies, *L*. *reuteri* I5007 was found to decrease the mRNA expression of the pro-inflammatory cytokine IL-1β in the ileum of neonatal piglets [14]. In contrast, TGF-β, which has generally been regarded as mediating immune tolerance and preventing excessive immune responses [50], had an increased expression in the mesenteric lymph node after treatment with *L*. *reuteri* in the every 4^th^ day treatment. Taken together, these data suggest that one explanation for the improved performance noted in previous studies with piglets treated with *L*. *reuteri* [14] could be a mitigation in the magnitude of immune responses thus allowing a greater proportion of the nutrients contained in feed to be directed towards growth rather than the immune system.

Probiotics have been widely studied in humans and animals. However, many questions regarding the use of probiotics remain to be answered. There is very limited information available about appropriate dosing regimens [51].

A previous study conducted in our laboratory showed that oral administration of *L*. *reuter*i I5007 improved suckling pig performance. However, in that study, *L*. *reuteri* was administered daily to suckling piglets for the first 14 days of life [14]. From a management perspective, this is very labor intensive. It is not known whether treatment for a shorter period of time or use of an intermittent treatment regimen can be effective in establishing an effective gut ecosystem. In the present study, differing durations of treatment (first 4-day treatment and every 4^th^ day treatment) of *L*. *reuteri* I5007 on gut microbial succession as well as immunological consequences in neonatal piglets were assessed. Based on the lower pH and lactic acid concentrations in the colonic digesta on day 14 as well as the increase in the relative abundance of mRNA for TGF-β and the decreased relative abundance of mRNA for IFN-γ observed in the mesenteric lymph nodes of piglets treated with *L*. *reuteri* every 4^th^ day, we suggest that prolonged treatment using the every 4^th^ day regimen is superior to treatment only during the first 4 days.

A shortcoming of the present study was that only one negative control group was used in the present study design with administration of 0.1% peptone water from day 1 to 4 while there was no similar control group for the pigs in the every 4^th^ day treatment. The lack of a second control group was a conscious decision due to lack of space on the gels and the general intensity of the work. It was also assumed that the placebo of 0.1% peptone would not affect intestinal microbiota succession and immunomodulatory consequences. However, animals in all three treatments were the same age and weight and the difference in pepton-placebo administration was the only confounding factor in the experiment.

In conclusion, early intervention with *L*. *reuteri* I5007 increases the presence of beneficial bacterial species and decreases the presence of undesirable microbes. The changes appear to be mediated by altering the environmental pH through lactic acid production resulting in favorable bacterial species colonization. The changes observed in the levels of the mRNA expression for IFN-γ and TGF-β in the mesenteric lymph nodes suggest that one explanation for the improved performance noted in previous studies with piglets treated with *L*. *reuteri* could be a reduction or prevention of an excessive immune response thus allowing a greater proportion of the nutrients contained in feed to be directed towards growth rather than the immune system. A prolonged duration of treatment (i.e. every 4^th^ day) would appear to be superior to treatment only during the first 4 days of life.
